# The reporting and handling of missing data in genetic epidemiological studies of mental health in childhood and adolescence: A systematic review

**DOI:** 10.1002/jcv2.70101

**Published:** 2026-02-27

**Authors:** Meseret M. Bazezew, Adrian Dahl Askelund, Kate Tilling, Alexandra Havdahl, Laurie J. Hannigan

**Affiliations:** ^1^ Research Department Lovisenberg Diaconal Hospital Oslo Norway; ^2^ PsychGen Centre for Genetic Epidemiology and Mental Health Norwegian Institute of Public Health Oslo Norway; ^3^ Department of Psychology University of Oslo Oslo Norway; ^4^ Population Health Sciences Bristol Medical School University of Bristol Bristol UK; ^5^ MRC Integrative Epidemiology Unit Bristol UK

**Keywords:** children and adolescents, genetics, mental health, missing data, polygenic scores, selective attrition

## Abstract

**Background:**

Genetic epidemiological analyses of child and adolescent mental health often use data from prospective longitudinal cohorts. Missingness due to selective attrition is therefore an important potential source of bias in such analyses. Informatively reporting on missingness and taking appropriate steps to handle it in analyses can mitigate this potential bias. Here, we aim to systematically assess how researchers report and address missingness in genetic epidemiological studies of child and adolescent mental health‐related outcomes using cohort data.

**Methods:**

We systematically searched the Ovid Medline database for studies published between August 2012 and August 2025, reporting polygenic score, genome‐wide association, or Mendelian randomization analyses, of data on children or adolescents participating in cohort studies. We extracted information from eligible studies based on criteria adapted from the strengthening and reporting of observational studies in epidemiology (STROBE) guidelines.

**Results:**

A total of 133 eligible studies were included, of which 125 (93.98%) reported the number of complete cases in all waves, while 84 (63.16%) detailed the amount of missingness on all key variables. Most studies used complete case analysis, while 39 studies explicitly reported applying other methods to handle missingness, with multiple imputation (*n* = 20, 15.04%) being the most common, followed by full information maximum likelihood 10 (8.1%). Only 18 studies (13.53%) reported an assumed missing mechanism along with the method used to address missingness. Full reporting of both the extent and handling of missingness at the item level was rare, occurring in only 5 (3.76%) and 15 (11.28%) studies, respectively, among the 123 studies that used multi‐item instruments.

**Conclusion:**

Best practice recommendations for reporting on missing data handling emphasize the importance of detailing the proportion of missingness, types of mechanisms underpinning missingness, and details of approaches used. Based on this review, these recommendations for proper reporting of missing data are rarely followed in full.

## Key Points

**What's known?**

- Missing data is ubiquitous in research and, if not handled appropriately, can distort the results of studies. For studies that rely on long‐term follow up over a number of years, this risk is particularly high.

**What's new?**

- Most researchers in the field of child and adolescent psychiatric genetic epidemiology using population‐based cohort data reported how much complete data was available for their analyses. However, many did not explicitly report the methods applied to handle missing data, and most failed to comprehensively report on the finer details their handling of missing data in accordance with guideline recommendations.

**What's relevant?**

- Future work should ensure a high standard of reporting and handling of missingness to ensure the validity and robustness of research findings.

## BACKGROUND

A notable methodological concern in longitudinal cohort study designs is the potential for participant attrition (Young et al., 2006)—where participants leave the study and do not return. Failing to appropriately address the issue of missing data due to attrition undermines the validity of research; not only by causing a reduction in statistical power and precision, but also by introducing the potential for bias in estimates and limiting the generalizability of findings (Deeg, 2002). Attrition often occurs selectively, which results in differences between the characteristics of those who remain in the study and those who do not. If these differences in characteristics are reflected in the outcome or exposure of a given analysis, it creates the potential for bias (Dziadkowiec et al., 2020; Gustavson et al., 2012; Kang, 2013; Little & Rubin, 2019; Schafer & Graham, 2002).

This is similar to bias caused by non‐randomised/selective participation *into* the cohort, but a critical distinction is that attrition‐related biases can often be mitigated by strategies that make use of observed data.

Research in the field of psychiatric genetic epidemiology, where longitudinal cohort studies are commonly used data sources, is highly vulnerable to missing data problems. Many commonly studied outcomes and exposures in psychiatric genetic epidemiological analyses have been shown to be associated with attrition, including individual characteristics such as social and lifestyle characteristics and psychiatric conditions and traits (Lamers et al., 2012; Taylor et al., 2018). Directly measured genetic factors in the form of polygenic scores (PGS) have also been shown to associate with attrition—for example, in the follow‐up waves of the Avon Longitudinal Study of Parents and Children (Taylor et al., 2018). Links between sociodemographic characteristics, psychiatric problems, and genetic liabilities captured by polygenic scores and attrition in cohort studies emphasize the need for genetic epidemiological analyses to mitigate the risk of bias by appropriately accounting for missingness.

Attrition can be minimized at different stages of the research, with strategies including selecting appropriate study designs, participant follow‐up techniques, and delivering specific training to those involved with recruitment and retention of participants in the study (Randomized Trials in Orthopaedic Surgery; Wilcox et al., 2001; Little et al., 2012). However, missing data is virtually inevitable in cohort designs. Consequently, handling missing data problem at the analysis stage becomes common. As a result, it is highly recommended to collect auxiliary information that can help predict missing values and allow for effective statistical adjustment and handling of missing data at the analysis stage (Little et al., 2024).

The effectiveness of approaches to address missing data at the analysis stage depends on several factors: the extent of missingness, which variables (outcome or exposure or both) are missing, the pattern of missing data (refers to which values in the data set are observed and which are missing), the assumed missing mechanisms (the reasons why values are missing), and the selection of suitable statistical methods (Little et al., 2024; Little & Rubin, 2019). As there is no set cutoff for an acceptable percentage of missing data in statistical analysis, researchers should not focus solely on the amount of missing data to decide whether to handle missingness, or the type of method to use (Madley‐Dowd et al., 2019; Schafer, 1999; Schafer & Graham, 2002). Missingness in either exposures or outcomes, as opposed to covariates, is most clearly indicative of the *potential* for bias in an analysis. However, whether specific estimates will *actually* be biased depends upon both the statistical model and the mechanisms underlying the missingness—which may differ for the different variables (Hughes et al., 2019; Madley‐Dowd et al., 2019; Schafer & Graham, 2002). Therefore, selecting the most appropriate method to account for missing data should depend upon an active consideration of each of these elements.

There are various methods to handle missing data in analyses of cohort data. Examples include *ad hoc* approaches such as mean imputation, last observation carried forward (LOCF), complete case analysis (CCA); principled methods such as multiple imputation (MI), full information maximum likelihood estimation (FIML); and weighting strategies (Little et al., 2024; Wilcox et al., 2001; Young et al., 2006). However, no single method is universally appropriate for all missing data scenarios, and much literature exists that explores and compares these approaches to missing data handling (Hughes et al., 2019; Little et al., 2024; White & Carlin, 2010; Zhu, 2014). Here, we briefly (and non‐exhaustively) summarise what is a highly nuanced picture. *Ad hoc* methods such as LOCF and mean imputation are generally avoided due to their risk of introducing bias in longitudinal data analysis irrespective of the missingness mechanism involved (Lachin, 2016; Schafer & Graham, 2002). Complete case analysis, FIML, and MI approaches provide unbiased estimates when the data are Missing Completely at Random (MCAR)—where missingness is unrelated to the observed or unobserved data (Little et al., 2024; Zhu, 2014). In linear regression analyses, MI may be preferable for reasons other than bias reduction, such as increasing the precision of estimates (Rubin, 1987; White & Carlin, 2010). Where missingness is systematic but explicable by observed data only (Missing at Random: MAR), CCA, FIML, and MI can all yield unbiased results depending on the combinations of missing data mechanisms involved (Little et al., 2024; Zhu, 2014). There are conditions in which CCA can represent an optimal approach compared to MI (Madley‐Dowd et al., 2019; White & Carlin, 2010). However, both MI (Schafer & Graham, 2002; White & Carlin, 2010) and FIML (Enders, 2022; Schafer & Graham, 2002; Tang & Tong, 2023) can, if correctly implemented, provide unbiased estimates in all situations of MAR in most statistical models. Weighting strategies, such as inverse probability weighting (IPW), may also be used to handle missingness under MAR, but are typically somewhat inefficient (Little et al., 2024).

The most challenging scenarios for researchers often involve handling missingness that are Missing Not At Random (MNAR)– where the missingness is explained by unobserved data. If missingness is in a univariate exposure or covariate under MNAR while all other variables in the model are complete, CCA and FIML provide unbiased estimates where MI may give biased results (Hughes et al., 2019; Madley‐Dowd et al., 2019; Schafer & Graham, 2002). However, if missingness is MNAR and occurs in the outcome and in more than one covariates/exposure, none of the established approaches are consistently unbiased. A few likelihood and MI‐based methods, with specific adjustment in the imputation model, have been proposed that show better performance to handle MNAR outcomes (Allison, 2012; Buuren & Groothuis‐Oudshoorn, 2011; Little et al., 2024). However, until now these methods have been rarely used, potentially because of their complexity, the unavailability of detailed guidance, and their requiring commercial software to apply (Allison, 2012; Little & Rubin, 2019; Rubin, 1987). Conducting sensitivity analysis assuming different missingness mechanisms is the most common way to assess whether assuming different missingness mechanisms impacts the conclusions drawn (Leurent et al., 2018). Similarly, across all scenarios, it may be advisable for researchers to perform more than one method, where feasible, and compare the results to assess robustness.

There are some additional considerations for evaluating missing data handling in longitudinal analyses. Studies show that multilevel models for repeated measures efficiently account for missing data if it occurs in the *outcome* variable under the MAR assumption (Verbeke & Molenberghs, 2001; van Buuren, 2018). In such models, the missing values of a variable Y are predicted based on the intermediate values of Y measured prior to drop‐out (and after intermittent missingness). However, in cases of MNAR or missingness in exposures/covariates or high level of missingness, other principled techniques may be necessary to reduce bias and improve estimates (Little et al., 2024; Verbeke & Molenberghs, 2001; van Buuren, 2018). When using MI, the imputation model should be specified correctly to get valid estimates—including accurately reflecting the data structure (e.g., a multilevel model for hierarchical data), compatibility of main analysis model and the imputation model (e.g., interactions in the main model should also feature in the imputation) and finally the inclusion of auxiliary variables in the imputation model (Schafer, 1999; Schafer & Graham, 2002; Zhu, 2014). A recent study showed how different amounts of missing data and the inclusion of auxiliary variables together influence the validity of estimates with MI (Madley‐Dowd et al., 2019).

Although attrition over time is a major source of missingness in cohort studies, data can also be missing in a more granular way. Many of the variables used in mental health research from population‐based cohorts are multi‐item instruments (i.e., a measure of one construct with multiple items/questions). In this case, missingness might occur at the item level. However, it has been shown that researchers give less attention to addressing missingness at the item level (van Ginkel et al., 2010). A study comparing various methods for handling missing data at the item level versus the total score level found that mean imputation was the least effective method for total scores regardless of the amount of missingness (Eekhout et al., 2014). Applying MI at the item level outperformed mean imputation by providing much more accurate coefficient estimates and standard errors, especially when a significant number of subjects had missing items surpassing 25% compared to applying to the total score. Overall, MI outperforms *ad hoc* methods and can produce minimal bias in model estimates for missing values in both total and item scores (Eekhout et al., 2014). However, most researchers might not be cognizant of accessible methods that are both simple and statistically superior, and designed for addressing missing data at the item level (van Ginkel et al., 2010).

Appropriate handling of missing data in genetic epidemiological analyses of cohort data by selecting and properly implementing suitable statistical methods—at both the variable and item levels—represents an important obligation for researchers seeking valid insights into their traits and mechanisms of interest. However, even the most well‐conducted analyses can be undermined by a lack of comprehensive and detailed reporting on the extent and handling of missingness. Practical frameworks for reporting the extent and handling of incomplete data in observational studies (Lee et al., 2021) are steadily advancing. The Strengthening the Reporting of Observational Studies in Epidemiology (STROBE) initiative developed recommendations on what should be included in an accurate and complete report of an observational study (Von et al., 2007). Moreover, there are useful additions to this framework that provide detailed instructions on how to report on missing data. Stern and colleagues (Sterne et al., 2009) provide general recommendations for the reporting of missing data and specific recommendations for reporting the details of MI (see also the framework for the Treatment and Reporting of Missing data in Observational Studies [TARMOS] (Lee et al., 2021)). These guidelines suggest that observational studies should include details regarding the extent of missing data, explanations for non‐participation and non‐response, the approach to addressing missing data in the analysis, assumptions regarding missing data mechanisms, and descriptions of any sensitivity analyses conducted. However, many of these details remain absent from reports of observational research.

Many systematic reviews have been conducted to assess how researchers are reporting and handling missing data in clinical and epidemiological longitudinal studies. Typically, these have shown consistently poor reporting including substantial variability in the standards of reporting and handling of missing data and inappropriate handling of missing data in longitudinal observational studies (Desai et al., 2011; Hunt et al., 2021; Karahalios et al., 2012; Lieber et al., 2020; Malhotra et al., 2019; Okpara et al., 2022; Powney et al., 2014; Richter et al., 2019; Wood et al., 2004). However, no previous review has focused on how researchers in the sub‐field of psychiatric genetic epidemiology report and handle missing data. While problems with handling and reporting of missing data are not exceptional to the field of psychiatric genetic epidemiology, reliance on cohort data and the evidence of common genetic liabilities being linked to attrition make potential bias due to selective attrition a serious risk for research in this field. Therefore, the aim of this study is to systematically assess and characterize how the reporting and handling of missing data has been conducted in psychiatric genetic epidemiological studies of mental health in childhood and adolescence based on the STROBE guidelines (Von et al., 2007) and recommendations in Sterne et al. (2009).

## METHODS

### Data sources and search strategy

We searched in the Ovid Medline database for psychiatric genetic epidemiological studies published as research papers or preprints from 1 August 2012 to 19 August 2025 in English language. This database additionally includes records in PubMed that do not feature in Medline, as well as preprints and other non‐indexed citations (see Ovid Medline 2025 Database Guide). The inclusion criteria were designed to capture studies that applied various genetic epidemiological methods such as PGS, genome‐wide association studies, and Mendelian randomization to assess child and adolescent mental health using cohort data. Any reviews or meta‐analytic studies were excluded.

We note that this review was not preregistered due to its having arisen from a smaller literature search for a PhD project. However, in order to maximise our transparency despite this restriction, we have made the searching terms, the data extraction checklist, and the extracted data on which the conclusions of the study are based publicly available at https://osf.io/zfcm4/.

The search strategy was developed in consultation with researchers working in the field of child and adolescent psychiatric genetic epidemiology. Key concepts from the intended scope were identified and translated into database‐specific keywords. Discussions mainly focused on which genetic epidemiological methods, child and adolescence mental health conditions, and what study population to focus on in order to calibrate the scope of this review. For example, a decision to prioritise the methods most commonly used in genetic epidemiological studies at the time the review was carried out led to the exclusion of non‐molecular genetic methodologies, such as twin and family studies, despite their obvious contributions to the genetic epidemiological literature. In addition to a wide array of specific mental health outcomes, we also opted to include the general term “mental health/illness/disorders” as a keyword to ensure a comprehensive list of studies addressing any mental health‐related traits and conditions in children and adolescents. The detailed search strategy can also be found in Appendix S1.

### Methods of the review

Initial inclusion and exclusion of studies was carried out by MMB. This process involved reviewing titles and abstracts for alignment with the stated criteria. The resulting list was reviewed by LJH, with resolution of cases where criteria were adjudged to be partially fulfilled by mutual discussion and review of full texts where necessary. Of the included studies, MMB reviewed 67 papers while 30 and 36 papers were reviewed independently by ADA and LJH respectively, with a random sub‐sample of these re‐reviewed by MMB. Finally, LJH reviewed a random sub‐sample of papers initially reviewed by MMB. Any uncertainties on the decision of inclusion of papers or during data extraction were resolved by discussion among these researchers. The review was carried out using a data extraction checklist (Table S1) developed based on the STROBE guidelines. The level of agreement between screeners was high due to the relatively objective nature of the extraction checklist, was restricted to situations where authors' wording was subject to some interpretation, and disagreement invariably involved criteria that were marked as either fulfilled or “partially” fulfilled (see Table S1 for specific criteria). Additional tables and methods sections from the journal website were checked for content relevant to the data extraction checklist if referred to in the articles.

This systematic review was conducted in accordance with PRISMA guidelines.

## RESULTS

### Study selection

**FIGURE 1:**
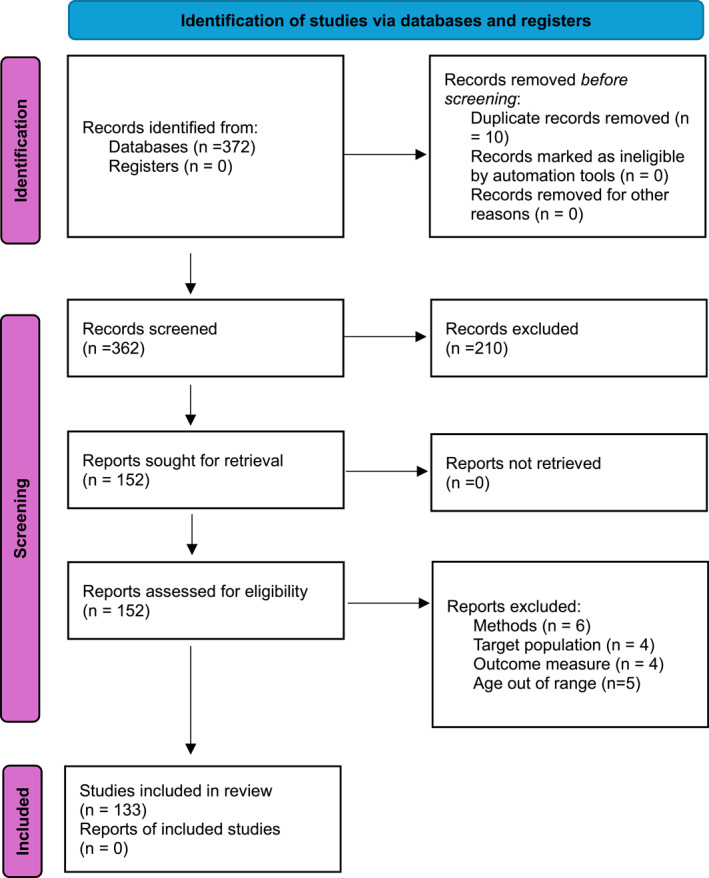
A flowchart of study inclusion process—PRISMA Chart.

A total of 372 journal articles were identified via the application of the search strategy to titles and abstracts in the OVID Medline database as of August 19, 2025. Based on title and abstract review 162 papers were identified for further evaluation. Of those papers, 10 papers were identified as duplicates. After full text review, 19 articles were excluded leaving 133 articles (Abdulkadir et al., 2023; Agnew‐Blais et al., 2021; Agnew‐Blais et al., 2022; Aguilar‐Lacasaña et al., 2022; Askeland et al., 2022; Askeland et al., 2023; Babineau et al., 2023; Barker et al., 2018; Bolhuis et al., 2022; Chang et al., 2020; Chen et al., 2020; Coleman et al., 2017; Cordova et al., 2022; Costantini et al., 2023; Diemer et al., 2023; Easey et al., 2021; González‐Peñas et al., 2020; Groen‐Blokhuis et al., 2014; Haan et al., 2021; Haan et al., 2022; Halldorsdottir et al., 2019; Hannigan et al., 2021; He and Li, 2022; Hosang et al., 2022; Hughes et al., 2022; Jones et al., 2022; Jones et al., 2018; Jones et al., 2019; Jones et al., 2016; Koomar et al., 2021; Lee et al., 2022; Liuhanen et al., 2023; Lussier et al., 2021; Ly et al., 2022; Ma et al., 2021; Machlitt‐Northen et al., 2022; Madley‐Dowd et al., 2022; Martel et al., 2022; Martin et al., 2014; Menta et al., 2023; Mills et al., 2017; Mistry et al., 2019; Morales‐Muñoz et al., 2023; Morneau‐Vaillancourt et al., 2021; Mullola et al., 2021; Musci et al., 2019; Musci et al., 2016; Nelemans et al., 2021; Newbury et al., 2022; Nilsen Husebye et al., 2023; Nivard et al., 2017; Paksarian et al., 2020; Perret et al., 2023; Perry et al., 2020; Pesonen et al., 2019; Pingault et al., 2023; Ponsonby et al., 2020; Price et al., 2020; Rahman et al., 2021; Rai et al., 2018; Ravagnani Salto et al., 2021; Reed et al., 2017; Rice et al., 2019; Riglin et al., 2017a; Riglin et al., 2016; Riglin et al., 2017b; Riglin et al., 2019a; Riglin et al., 2019b; Riglin et al., 2020; Riglin et al., 2022; Robinson et al., 2020; Sadik et al., 2022; Schellhas et al., 2021; Schlag et al., 2022; Serdarevic et al., 2020; Shao et al., 2021; Solmi et al., 2020; Solmi et al., 2019; Speyer et al., 2022; Stergiakouli et al., 2015; Sudre et al., 2021; Takahashi et al., 2020b; Takahashi et al., 2020a; Takahashi et al., 2023; Takahashi et al., 2022; Tanner et al., 2022; Thng et al., 2022; Thompson et al., 2020; Tobarra‐Sanchez et al., 2022; Velthorst et al., 2018; Vuijk et al., 2020; Wang et al., 2020; Warrier et al., 2021; Yang et al., 2022; Zammit et al., 2014) (Abou Assi et al., 2025; Allegrini et al., 2025; Askelund et al., 2024; Birkenæs et al., 2025; Campbell et al., 2025; Chen et al., 2024; D’Urso et al., 2024; Elagali et al., 2025; Espinosa Dice et al., 2025; Freitag et al., 2025; Grimes et al., 2024; Gu et al., 2023; Hernández‐Lorca et al., 2023; Huang et al., 2024; Karcher et al., 2025; Kunitoki et al., 2023; Misztal et al., 2023; Mooney et al., 2023, 2025; Nguyen et al., 2023; Pang et al., 2025; Power et al., 2025; Ravagnani Salto et al., 2025; Schiavon et al., 2025; Shakeshaft et al., 2025; Socrates et al., 2024; Sun et al., 2024; Takahashi et al., 2025; Teeuw et al., 2023; Tesli et al., 2024; Tharaud & Nikolas, 2025; Vincenzo et al., 2024; Wang et al., 2024; Xu et al., 2025; Zhang et al., 2023; Zheng et al., 2025) that met the eligibility criteria (see Figure 1 and online repository, https://osf.io/zfcm4/).

Table 1 shows the characteristics of the 133 studies that were included in the review. Most (79.69%) of the included studies were published within the last six eligible years (i.e., since 2020), reflecting our focus on contemporary genetic epidemiological methods. The most common area of study, comprising 42.86% of the included studies, was neurodevelopmental conditions and related traits, followed by general mental health problems (18.05%) and mood and anxiety disorders (14.2%; Table 1).

**TABLE 1:** Characteristics of reviewed papers.

Characteristics of reviewed papers	*n* (%)
Year published	
2014	3 (2.25)
2015	1 (0.75)
2016	3 (2.25)
2017	6 (4.51)
2018	5 (3.76)
2019	9 (6.77)
2020	16 (12.03)
2021	17 (12.78)
2022	25 (18.79)
2023	19 (14.28)
2024	12 (9.02)
2025	17 (12.78)
Total	133 (100)
Area of study	
Neurodevelopmental conditions and related traits	57 (42.86)
Mood and anxiety disorders, and related traits	19 (14.28)
Mental health (general)	24 (18.05)
Behavioural problems	12 (9.02)
Schizophrenia and psychotic symptoms	12 (9.02)
Eating disorders and related traits	4 (3.01)
Bipolar	3 (2.25)
Sleep	1 (0.75)
Suicidality	1 (0.75)
Total	133 (100)

### Reporting of missing data

Of the 133 studies included, 125 (93.98%) reported the number or proportion of complete cases (or, conversely, the proportion of missingness) at all relevant data collection waves. Furthermore, 84 (63.16%) studies clearly reported complete cases or number of missing on all main variables of interest at all waves (Table 2). Only just over a third (35.34%) of studies performed further investigation of the missingess by comparing complete and incomplete data on at least one key variable. Of these, 40 (85.1%) clearly reported the formal statistical test they used to compare (Table 2).

**TABLE 2:** Reporting of missing data and comparison of complete and incomplete data.

Reporting of missingness	*n* (%)
Number or % of missingness on individuals/cases at all waves	
Yes	125 (93.98)
No	8 (6.02)
Total	133 (100)
Number or % of missingness on key variables at all waves	
Yes	84 (63.16)
No	47 (35.34)
Partial	2 (1.5)
Total	133 (100)
Explicit comparison of completed and incomplete data	
Yes	47 (35.34)
No	84 (63.16)
NA	2 (1.5)
Total	133 (100)
Formal statistical test used to compare	
Yes	40 (30.08)
No	7 (5.26)
NA	86 (64.66)
Total	133 (100)

### Multi‐item instrument reporting and handling of missing data

Among the 123 studies that employed multi‐item instruments, only five (4.06%) reported the amount of missing data at the item level. Even though most of the studies did not report the amount of missingness at the item level, 15 (12.19%) studies explicitly outlined their strategies for addressing missingness at the item level, while 6 (4.88%) of them partially reported the handling strategies (Table 3). Some of the strategies outlined to handle missingness at the item level involved the exclusion of participants with >30% missing items, mean imputation when less than 30% items were missing, and the calculation of prorated summary scores for individuals with at least 80% of item‐level information available.

**TABLE 3:** Reporting of multi‐item instruments missingness and handling.

Multi‐item instruments	*n* (%)
Multi item instrument used	
Yes	123 (92.48)
No	10 (7.52)
Total	123
If multi‐item measures were used‐ *n* or % of missingness in single/each item reported *n* = 123	
Yes	5 (4.06)
No	118 (95.94)
Reporting handling of item level missingness *n* = 123	
Yes	15 (12.19)
No	102 (82.92)
Partial	6 (4.88)

### Handling of missing data

The assumed missing mechanisms were explicitly reported by 18 (13.53%) of the studies, along with the method used to handle missingness. Of these, most reported the assumed missing data mechanism for only one variable, despite missing data on other variables. Only two studies explicitly stated the same mechanism assumed for both the exposure and the outcome. Five studies reported the assumed mechanism for the outcome only, and seven reported for the exposure only.

Seventeen studies (12.78%) explicitly reported that they used CCA to handle missingness (Table 4). Of these, most did so by clearly stating, when describing the analyses, that they restricted to those in the sample with complete data. In one study, the authors explicitly mentioned that CCA was used as the strategy to handle missingness because of the assumed missing mechanism MCAR. Seventy‐seven studies (57.89%) did not explicitly report any methods applied to account for missingness. Consequently, since these studies did have missing data, it was inferred that CCA was used (the rationale for this inference is discussed in full in the Discussion section).

**TABLE 4:** Reporting of the main features of handling of missingness.

Reported missingness handling	*n* (%)
Missing mechanism assumed	
MAR^a^	14 (10.53)
MCAR^b^	2 (1.5)
MNAR^c^	2 (1.5)
Not reported	115 (86.46)
Total	133 (100%)
Type of analysis used to account for missingness	
Hot deck	1 (0.75)
Random forest	1 (0.75)
MI^d^ and FIML^e^	1 (0.75)
IPW^f^	5 (3.76)
FIML^e^	10 (7.52)
CCA^g^	17 (12.78)
MI^d^	18 (13.53)
CCA Assumed	77 (57.89)
Item level pairwise deletion	1 (0.75)
MI and IPW	1 (0.75)
Single imputation (any)	1 (0.75)
Total	133 (100%)

^a^ Missing at random.

^b^ Missing completely at random.

^c^ Missing not at random.

^d^ Multiple imputation 5.

^e^ Full information maximum likelihood.

^f^ Inverse probability weighting.

^g^ Complete case analysis.

The remaining 39 (29.33%) studies applied a missing data handling technique other than CCA. Applied techniques included MI (18; 13.53%), FIML (10; 7.52%), Inverse Probability Weighting (IPW; 5; 3.76%), Hot deck (1; 0.75%), a random forest method (1; 0.75%), both FIML and MI together (1; 0.75%) and IPW and MI together (1; 0.75%) (Table 4). Of these 39 studies, 14 studies did not perform a comparison between complete and incomplete data on key variables while three studies that used FIML and random forest, did not report the amount of missingness in their data. Approximately half of the studies that applied FIML and more than one third of studies that applied MI, five (50.0%) and seven (39.0%) respectively, described the assumed missing mechanisms (Table 5).

**TABLE 5:** Count of missing mechanisms reported along with missing data handling method.

Type of analysis used to account for missingness	MAR	MCAR	MNAR	Not reported	Total
CCA	0	1	0	17	17
FIML	5	0	0	5	10
Hot deck	1	0	0	0	1
IPW^a^	0	0	2	3	5
MI	6	1	0	11	18
MI and FIML	1	0	0	0	1
CCA assumed	0	0	0	77	77
Random forest	0	0	0	1	1
Item level pairwise deletion	1	0	0	0	1
MI and IPW	0	0	0	1	1
Single imputation (any)	0	0	0	1	1
Total	14	2	2	115	133

^a^ Inverse probability weighting.

### Multiple imputation

In accordance with our extraction checklist, we additionally reviewed specific elements of MI reporting among those studies that used MI to handle missingness. Out of the 20 studies that used MI (including two studies using both MI & FIML and MI & IPW), eight (40.0%) reported the assumed missing mechanism and 12 (60.0%) conducted a comparison between individuals with complete and incomplete data based on other characteristics in their analytic dataset. Except for two studies, all reported the software and package used for MI (and all used the *mice* package in R (Buuren & Groothuis‐Oudshoorn, 2011) or Stata). Fifteen (75%) of the studies reported the number of datasets imputed, while 11 (55%) explicitly reported the substantive and auxiliary variables included in the imputation model with two studies partially meeting this criterion (Table 6).

**TABLE 6:** Detail of multiple imputation out of 20 studies.

	*n* (%)
Reported features of studies applied MI	
Software and package	18 (90%)
Number of imputed data sets	15 (75%)
Explicitly reported substantive and auxiliary variables included	11 (55%)
Specification of the imputation model, including interaction terms, multilevel structure, link function	5 (25%)
Validity of imputed datasets assessed	4 (20.0%)
Sensitivity analysis of the MI results	11 (55%)
Reported software and package detail	
MICE^a^ package in R	9 (45%)
MICE package in STATA	7 (35%)
MICE package, software not reported	2 (10%)
Not reported	2 (10%)

^a^ Multiple imputation by chained equation.

The specification of the imputation model, including interaction terms, multilevel structure, or link functions, was clearly reported by only five (25%) studies. Four (20.0%) studies reported assessing the quality of the imputed datasets (comparing the distribution of observed and imputed datasets) and 11 (55%) performed sensitivity analysis to assess the MI estimates.

### Selection bias

Given the nature of the datasets used in the included studies—that is, population‐based cohorts reliant on voluntary initial participation—our extraction checklist also included several criteria to assess whether missingness due to *initial* selection into the cohorts was mentioned or examined. Seventy‐one (53.38%) studies referenced selection into the cohorts as a potential source of bias. Of these, 59 (83.09%) studies clearly reported on the potential for bias by providing information about differences between the sample and the target population. These differences were explicitly quantified in 29 (40.84%) studies. Twelve (16.90%) studies partially fulfilled these criteria (e.g., by noting the potential for bias caused by differences between a sub‐cohort and full cohort).

## DISCUSSION

In this systematic review, we identified and extracted information about the reporting and handling of missing data from 133 studies in the field of child and adolescent psychiatric genetic epidemiology. Despite high prevalence of reporting of the number of complete cases (or proportion of missing data), less than half (56; 42.11%) of the total studies in this review explicitly provided information about the methods used to handle missingness and only 18 (13.53%) reported the assumed missing mechanism along with the method they used. A majority of studies used CCA 94 (70.67%), but more often than not this was inferred rather than explicitly stated.

Almost all included studies performed descriptive analysis as the first step in their data analysis, which is important to understand the extent of missing data (Okpara et al., 2022). Consequently, the number or proportion of complete cases was easily identified, and the reporting of this was found to be common. This result aligns with a previous review study (Karahalios et al., 2012) where 80% of studies reported the amount of missing data. However, the prevalence of reporting the amount of missing data on all key variables, particularly in longitudinal studies, were lower. On the other hand, while most studies provided information about the number of complete cases, more than half 77 (57.89%) of studies did not comment beyond the presence of missing data on how it was handled, necessitating our assumption that CCA was used (more detail on this assumption is provided below).

Despite the widespread use of multi‐item instruments in psychiatric genetic epidemiological studies, reporting about the extent and handling of item level missingness was observed to be substantially limited. Indeed, only a handful of studies among those reviewed here provided information on the extent of item‐level missingness (for example Batra et al., 2021; Caramaschi et al., 2018). Additionally, the studies that addressed item‐level or total score missingness often used methods such as excluding missing observations or mean imputation, both of which could potentially introduce bias (Eekhout et al., 2014). Although it is possible that this was a considered decision in some cases, since mean imputation is considered unbiased when reliability of the instrument is >0.70 and is recommended in such scenarios when the application of item‐level MI is impractical (Young et al., 2006), the fact that many of these studies do not even report the proportion of item‐level missingness may suggest otherwise. There are several possible reasons for the limited attention paid to item‐level missingness in the studies included in this review. Many cohorts provide researchers with pre‐computed scale variables, which may mean that those analysing the data have either not made the decisions about item‐level missingness or may even be unaware as to whether data were missing at the item‐level or may not have access to deal with it. Alternatively, pragmatic decisions to maximise computing resources (e.g., avoiding MI for many individual items, which is computationally intensive) may be behind the observed pattern. In some cases, researchers may simply have been unaware of the potential impacts of item‐level missingness on results. Nonetheless, some acknowledgement and assessment of the potential for bias from item‐level missingness could strengthen the reporting of analyses using multi‐item instruments.

Less than half of the studies (56; 42.11%) in this review provided information about the methods used to handle missingness. Of these studies, only a minority (18; 13.53%) provided information about the missing mechanism assumed. Assessment of assumed missing mechanisms have been found to be similarly uncommon in other reviews of observational studies by Richter and colleagues (Richter et al., 2019) and Okpara and colleagues (Okpara et al., 2022). The latter review article (Okpara et al., 2022) reported that only 11.3% of studies described the missing mechanisms assumed along with the methods used to handle missingness. Although it may be challenging to definitively establish the mechanisms causing missing data in a dataset, there are certain evaluations that can help inform our assumptions (Ibrahim et al., 2012). Comparing the baseline characteristics of individuals with complete data to those with incomplete data (e.g., using Little's MCAR test (Little, 1988)) can reveal whether the missing data is related to observed variables (Richter et al., 2019). If this test does not support the null hypothesis of no difference in baseline characteristics, the data is likely not MCAR (it could be MAR or MNAR). Prediction of missingness for a given variable by logistic regression on covariates may then provide further clarity on the extent to which MAR can be reasonably assumed (Fairclough, 2010). In addition, researchers usually have to draw on their knowledge of the study and its context to assess whether the assumed mechanism are appropriate (Hughes et al., 2019; Ibrahim et al., 2012). For example, if participants are hesitant to respond to sensitive questions, this could suggest that the missing data for those questions may follow MNAR mechanism.

In this review, due to a lack of explicit reporting, we inferred the use of CCA for 77 (57.89%) studies that noted having missing data but did not explicitly describe a missing data handling method (i.e., when authors gave a lower N for some predictor or outcome than the total sample size, and then proceeded to report analytic results for the same predictor or outcome). This number compares to only 17 (12.78%) studies that explicitly stated that they used CCA. While, in the absence of direct reporting, we cannot be entirely certain what approach was used we consider it a reasonable assumption that researchers using more “hands‐on” approaches to missing data handling such as MI would be very unlikely to fail to report this. Moreover, CCA is a default in the common software implementations of the regression‐based analyses that underpin many of the methods in the studies included in this review (e.g., in the *lm()* and *glm()* functions of the *stats* package in R). Therefore, we are confident in concluding that CCA is the most frequently used method to handle missingness, despite being *explicitly* reported less often than MI. The predominant lack of explicit reporting when researchers use CCA may indicate that it is used as a default option, a lack of awareness about alternative methods for handling missingness, or uncertainty about how to properly report missing data and handling techniques. While CCA may be an appropriate strategy to handle missing data without the introduction of bias in some scenarios (Hughes et al., 2019; Madley‐Dowd et al., 2019), readers should be informed about this decision and the logic behind it in order to make that evaluation. No missing data handling method can be safely treated as a default option, and careful consideration of the relevant factors should be accompanied by clear reporting, and explicit consideration of the potential impact of missingness on results in the context of the strategy employed.

Only a few studies 20 (15.04%) applied MI despite its availability in mainstream statistical software. Nonetheless, it was the most popular method among those studies that addressed missingness other than CCA, followed by FIML 10 (7.52%). In terms of the popularity of the methods other than CCA, our findings are similar to a previous review published in 2022 (Okpara et al., 2022) where 14.3% included studies used MI and 4.3% used FIML. The majority (13; 72.22%) of the studies in our review that reported on missing data mechanisms were from those that applied MI and FIML. This indicates that researchers who applied MI and FIML likely paid more attention to the underlying missing data mechanisms.

Even though existing guidelines recommend detailed reporting of MI procedures, many studies using MI provided limited information. Incomplete reporting can pose challenges in appraising a study's approach to handling missing data. Most studies presented the number of imputed datasets, the software and package used. However, assessment of the quality of the imputed datasets, and specifications of the imputation model, including interaction terms, multilevel structure, and link functions were not commonly presented. To ensure that the imputation process has been carried out effectively and that the resulting dataset accurately represents the missing values, it is recommended to evaluate the quality of the imputed datasets (Sterne et al., 2009). Indeed, some researchers may have done this as part of their MI analyses, but readers' confidence in their findings could be strengthened if the results of these evaluations were clearly reported.

Finally, it is unclear whether researchers in the field are aware of the link between selection bias and missingness due to attrition. That is, when eligible individuals not included in a sample differ systematically from those who are included (selection bias (Desai et al., 2011)), this essentially gives rise to MAR or MNAR scenarios similar to those arising when individuals who drop out of the study differ from those who remain involved. Many of the studies (59; 44.36%) included in this review at least referenced the possibility of bias due to selective participation in cohorts or cohorts' non‐representativeness of the target population—typically while discussing limitations of their results. Furthermore, in a variant of this issue, (6.02%) studies chose their sample from a cohort based on data completeness as eligibility criteria. Ultimately, researchers should be aware that non‐random participation in cohorts from recruitment and selective inclusion in analytic samples based on availability of data are both intrinsic sources of potential bias in observational research. Each of these must be attended to with care to preserve the validity and generalisability of results.

### Limitations

In this review, we included a substantial number of studies from across the field of psychiatric genetic epidemiology, with a focus on child and adolescent samples. There are some limitations to our approach. First, all the publications were required to be in English, which could limit the generalizability of our findings. Second, while data extraction was standardised and validated as far as possible, the broad range of different ways in which researchers can report on missingness, and missing data handling presented a challenge. It is likely that in some cases our assessments about the completeness of reporting could be contested by authors—perhaps in view of some small detail that was missed on our evaluation or some nuance of our criteria. In acknowledgement of this, we have not attempted to draw out specific examples of the various practices we observed, instead emphasising the patterns across studies, in which we can have more certainty. Third, more than half of studies, according to our criteria, were recorded as not having provided any information about the technique they used to handle missingness, and we opted to assume that those studies used CCA. This may have been incorrect in some cases—for example, in cases where researchers used mixed models, which can account for missingness in a specific way that does not equate to CCA. Fourth, although stating the reason for missingness is one of the main points in the STROBE guideline, we opted not to assess this in our review because our inclusion criteria (and focus on population‐based cohorts) meant that this was overwhelmingly likely to be attrition in most cases. Finally, in this review we did not differentiate studies that performed analysis on a single wave or multiple waves of data to present the reporting and handling of missing data techniques separately.

## CONCLUSION

Researchers using population‐based cohort data in the field of child and adolescent psychiatric genetic epidemiology did typically report the number of complete cases used in their analyses. However, most failed to comprehensively report on their handling of missing data in accordance with guideline recommendations. For readers to assess the risk of bias in results, it is crucial that they are provided with information about the amount of missing data, assumed missing mechanisms, and the way in which missingness was accounted for in analytic models.

Overall, more robust reporting—and, likely, more careful and considered missing data handling—is needed to ensure that researchers in this field can obtain unbiased estimates from their studies. Besides this, enhancing the standard of reporting and handling of missingness could contribute more broadly to the validity and reproducibility of research.

## AUTHOR CONTRIBUTIONS

**Meseret M. Bazezew**: Conceptualization; systematic search; identifying eligible papers; development of extraction criteria; data extraction; analysis; interpretation; writing—original draft; writing—review and editing. **Adrian Dahl Askelund**: Data Extraction, writing—review and editing. **Kate Tilling**: Interpretation; writing—review and editing. **Alexandra Havdahl**: Funding Acquisition; conceptualization; supervision; project administration; resources; writing—review and editing. **Laurie J. Hannigan**: Funding Acquisition; conceptualization; input on systematic search; development of extraction criteria; interpretation; writing—review and editing. All co‐authors read and approved the final manuscript.

## CONFLICT OF INTEREST STATEMENT

L.J.H. is a joint editor at JCPPA. The remaining authors have declared that they have no competing or potential conflicts of interest.

## ETHICAL CONSIDERATIONS

The work presented in this manuscript is a systematic review of published articles for which specific ethical approval was not required. It involves no analysis of participant data for which consent would be required.

## TRIAL REGISTRATION

The authors note that this review was not preregistered due to its having arisen from a smaller literature search for a PhD project. However, in order to maximise their transparency despite this restriction, they have made the searching terms, the data extraction checklist, and the extracted data on which the conclusions of the study are based publicly available at https://osf.io/zfcm4/

## Supporting information

Supporting Information S1

## Data Availability

The extracted dataset supporting the conclusions of this article is available at https://osf.io/zfcm4/.
