# Supplementary material for: The reporting and handling of missing data in genetic epidemiological studies of mental health in childhood and adolescence: A systematic review
Source: JCPP Adv. 2026 Feb 27:e70101. Online ahead of print. doi: 10.1002/jcv2.70101 (PMC13339435; doi:10.1002/jcv2.70101)
Supplement: Supplementary file 1 — Supporting Information S1 [file JCV2-9999-e70101-s001.docx]

***The reporting and handling of missing data in genetic epidemiological studies of mental health in childhood and adolescence: A systematic review***

## **Supporting Information**

## Appendix S1. Searching strategy: MS word

**Study design**: Birth-cohort or Cohort or Longitudinal or clinical cohort.

**Analysis methods**: Polygenic Score or Polygenic Risk or Genetic Risk Score or Genetic Score* or Genetic instrument or Mendelian Random*

**Mental health outcomes**: mental disorder or mental health or mental illness or mental problem or depress* or attention deficit hyperactivity disorder or attention-deficit hyperactivity disorder or autis* or psychiat* or bipolar or schizo* or psycho* or eating disorder* or anorex* or bulimi* or adhd or asd or mdd or anxiety or oppositional defiant disorder or conduct disorder or obsessive compulsive disorder or neurodevelopment* or ocd or post-traumatic stress disorder or posttraumatic stress disorder or post traumatic stress disorder or ptsd or tourette* or phobia)

**Population:** Child* or infant or adolescen* or school*).

**Study period**: 2012 – 2025

**Language*:*** English

| **Table S1. Data extraction checklist: MS word** | | |
| --- | --- | --- |
| General data extraction | | |
| REPORTING | 1. Do the authors explicitly report that there is no missing data? | ***If Y, stop here*** |
|  | 1. Do they report the number or proportion of missing or non-missing… |  |
|  | 2A) ... individuals/cases at all waves used in analysis?  *NB – this is* ***fulfilled*** *either if authors provide minimal information specifically relating to individual-level missingness (e.g., “42% of the sample had data available at wave X, if they only analyze at wave X,”) or if they provide variable-level missingness, fulfilling the criterion below) …* | ***Y/N/NA*** |
|  | 2B)...values on all variables of interest at all waves used in analysis. | ***Y/N/ NA*** |
|  | *2C) (if multi-item measures are used)* ...each item comprising variables of interest? | ***Y/N/NA*** |
|  | 1. (if multi-item measures are used) Do they report how they handle the missingness at the item level?   *Example 1: “Summary scores were calculated for individuals with at least 80% of item-level information.”*  *=Partial (listwise deletion + some unspecified approach)*  *Example 2:* “*Participants missing more than half the items were coded as missing for the scale; scale scores of participants missing less than half the items were prorated*.” *=Yes* | ***Y/N/Partial/NA*** |
|  | 1. Do they explicitly compare individuals with complete and incomplete data (on at least one key variable)?  - *Provided a table comparing distributions of key exposures and outcome variables for those with missing and non-missing information … Yes* - *Table not provided but some summary statistics included in text …yes* - *General comment provided (did not include a table or summary statistics or included p-values only) ….yes* | ***Y/N//NA*** |
|  | *4A) (If yes)* is a formal statistical comparison carried out? | ***Y/N/NA*** |
| HANDLING | 1. How are missing data handled in the main analyses?   *NB: for those studies that don’t apply or mention anything about missingness/method, NA will be recorded.*  *Not reported will be recorded if they mentioned that they handle missingness but the method is not clearly presented* | ***Complete case / Single imputation (any) / MI / ML / IPW / Other/NA/Not reported/***  ***Multiple approaches (list)*** |
|  | 1. What mechanism of missingness is assumed? | ***MAR / MCAR / MNAR / Not reported / NA*** |
| SELECTION | 1. Are differences between the analytic sample and the population from which the sample was drawn mentioned as a potential source of bias?   *NB – the most likely place for authors to mention this is in a discussion of the limitations of their study; look for mentions of ‘selection’, ‘generalisability’, ‘ascertainment bias’ or similar*  *If the comparison made is between the analytic (sub-)sample and the full cohort, this should be recorded as “Partial”*  *If the comparison made is between the cohort and the population, this should be recorded as “Y”* | ***Y/Partial/N*** |
|  | *7A) (If yes)* are differences between the sample and the population quantified (either directly, in the paper, or in an explicitly cited source)?  *Example 1: “MoBa participants have been shown to be significantly wealthier and healthier than the Norwegian general population (cited reference)” =Yes* | ***Y/N/NA*** |
|  | *7B) (If yes)* are any sensitivity analyses or formal adjustments (e.g., weighting) performed to correct estimates for possible selection bias?  *Record NA:-If they mentioned the sample is a good representation of the population* | ***Y/N/NA/NA*** |
| Additional data extraction for studies using MI only | | |
| MI | 8. What software/package is used for the imputation? | ***>Software/package details< / Not reported*** |
|  | 1. How many datasets were imputed? | ***Number/Not reported*** |
|  | 1. Are all variables used in the imputation clearly listed? | ***Y/N/partial*** |
|  | 1. If used, are auxiliary variables clearly identified as such?   *NB: We want to see if they explicitly mention auxiliary var by mentioning the var that has or is expected to have association with the missing var (variable of interest) included in the imputation model.* | ***Y/N/NA*** |
|  | 12. Is the specification of the imputation model clearly reported?  *We want to see if they report the details of MI model regardless of its correctness. If including interaction terms, specification of link functions, and multilevel structure if appropriate are reported)?*  *NB we cannot assess model misspecification with this criterion; the model could be incompletely reported but correctly specified, or reported in full but misspecified* | ***Y/N/partial*** |
|  | 13. Are any comparisons carried out to assess the validity of the imputation? (e.g., comparing observed/imputed values) | ***Y/N/NA*** |
|  | 14. Are any sensitivity analyses carried out to assess the impact on the study results of using imputed data (e.g., MI vs. CCA)? | ***Y/N/NA*** |
